# Impact of mode hybridization on spin-wave profiles in bi-component magnonic crystals

**DOI:** 10.1038/s41598-026-42425-y

**Published:** 2026-03-14

**Authors:** S. Mamica

**Affiliations:** ISQI, Faculty of Physics and Astronomy, A. Mickiewicz University in Poznań, ul. Uniwersytetu Poznańskiego 2, Poznań, 61-614 Poland

**Keywords:** Magnonic crystals, Spin-waves, Mode coupling, Hybridization, Multimode hybridization, Mixing of profiles, Materials science, Physics

## Abstract

The study investigates mode hybridisation in two-dimensional permalloy–cobalt magnonic crystals. Calculations were performed using the plane-wave method, with particular emphasis on the effects accompanying hybridisation. A distinctive feature revealed in this work is the exchange, between hybridising modes, of the excitation concentration coefficient in the constituent materials (e.g. in cobalt). This coefficient describes the fraction of spin-wave energy concentrated within one of the components of the composite. Its analysis enables the identification of hybridisation even when other manifestations—such as profile swapping or clear branch repulsion—are only weakly pronounced. We demonstrate cases in which the exchange of the concentration coefficient occurs between hybridising modes without the typical swapping of spin-wave profiles, or with only minimal repulsion between their branches in the frequency spectrum.

## Introduction

Magnetic materials host multiple competing interactions—such as exchange, dipolar, and Dzyaloshinskii–Moriya—which shape both their static and dynamic properties. Their interplay gives rise to complex equilibrium magnetisation configurations, including vortices^1–3^, labyrinth domains^4,5^, and skyrmions^6–11^. Of particular interest, however, are the dynamical consequences of these interactions, as they determine the spin-wave spectrum—collective excitations of the magnetisation—leading to a broad variety of important effects^12–17^. One example is the non-uniform shift of spin-wave frequencies under variation of the external magnetic field, observed in many magnetic systems^18–24^. As the field changes, certain modes approach one another in frequency. If their symmetries are compatible, they may couple and subsequently hybridise, typically manifested through branch anti-crossing (avoided crossing), mode-order interchange, and the mixing of spatial profiles^25–27^.

Spin-wave hybridisation is central to modern magnonic engineering. It enables precise control of wave dispersion and dynamics and underpins the operation of numerous functional elements—from filters and diodes to resonators and multiplexers, as well as coherent magnonic systems. Accordingly, the analysis of hybridisation and its physical and technological implications constitutes a key topic in contemporary magnonics research. Hybridisation allows controlled coupling between modes of different nature (e.g., dipolar and exchange, surface and volume, magnonic and acoustic), thereby enabling the formation of hybrid states with new, functionally relevant properties. Recent review articles highlight that strong coupling of spin waves and their hybridisation with other excitations form the basis of developments in wave-based processing, integration with quantum technologies, and reconfigurable magnonic architectures^28–30^.

A significant class of hybridisation-based applications involves the design of functional elements that control spin-wave propagation, such as diodes, circulators, directional couplers, and frequency-selective filters. Strong hybridisation of dipolar and exchange modes in thin films and multilayers enables, for instance, enhanced group velocities and selective damping of particular modes. In systems such as directional-coupling structures, nanomagnonic slow-wave devices, and multilayers with tunable interlayer coupling, hybridisation can be exploited to realise nonreciprocal functionalities or logic-type operations^31–39^.

Another rapidly growing research direction concerns resonator-type structures, where hybridisation gives rise to specialised eigenmodes such as dark modes or energy-localised states. In chiral resonators, hybridisation allows selective manipulation of spin-wave energy, and in nonlinear regimes it supports functionalities inspired by neuromorphic computing^35,40^. In two-dimensional materials and van der Waals magnets, the possibility of smoothly tuning coupling strength between modes has also been demonstrated^36,41^.

A particularly important development involves systems in which magnons strongly couple to other quasiparticles or fields, such as phonons, acoustic waves, photons, or optical modes. Spin-wave-excitation enhancement was observed due to the photon-magnon hybridisation^42^. Magnon–phonon (magnon-polaron) hybridisation enables extended coherent transport, amplification or suppression of selected dispersion branches, and the emergence of topological effects^43–46^. In acousto-magnonic structures, strong hybridisation facilitates tunable filtering and switching functionalities^47^. Furthermore, in coherent systems, hybridisation plays a crucial role in enhancing spin pumping and energy transfer between modes, including three-mode processes^48,49^.

Recent studies also show that spin-wave hybridisation is a key mechanism in designing reprogrammable magnonic systems. In ferrimagnetic heterostructures, dynamic reconfiguration of mode coupling has been achieved^50^. In multilayers and nanodomain systems, hybridisation of standing exchange modes, surface modes, and anisotropy-driven modes has been demonstrated to enable selective propagation channels and the engineering of complex spin-wave energy landscapes^51–53^.

Hybridisation plays a central role in magnonic crystals (MCs)^54,55^, the magnetic counterparts of photonic^56^ and phononic crystals^57,58^. One of its key consequences is the opening of band gaps in the spin-wave spectrum^59,60^. In bi-component MCs, an additional effect arises from the magnetisation contrast between the constituent materials: spin waves are excited with different intensities in each component. It has been shown that variation of the external magnetic field may induce a redistribution of excitation energy between the components of the composite^61^. Similar observations were made in systems where the spin-wave spectrum is shaped by cobalt dots only partially embedded in a permalloy matrix^62,63^.

Experimental observations of spin-wave hybridisation have also been reported in antidot lattices (ADLs). In^64^, two types of structures were compared: Py-based ADLs with alternating hole diameters and two-dimensional MCs with cobalt dots partially embedded in Py. In both cases, clear coupling between different spin-wave modes was observed. In ADLs, the variation in hole sizes led to strong hybridisation of localised modes, whereas in the 2D MC the mode localised at small wave vectors gradually delocalised into the matrix with increasing wave vector, directly reflecting hybridisation.

An interesting phenomenon was reported in^65^, involving an ADL fabricated from a Co/Pd multilayer, where the processing resulted in the formation of a ring with gradually varying magnetic parameters around each hole. Volume and ring-type modes were identified, and hybridisation between them occurred within specific external-field ranges. Conversely^66^, emphasised that even in the absence of such a ring, strong demagnetising fields arise near antidot edges, modifying local propagation conditions and giving rise to additional hybridisation pathways.

In our earlier work on spin-wave hybridisation in Co/Py MCs^27^, we showed that compressing the structure along the external-field direction shifts hybridisation of selected mode pairs towards higher fields. Moreover, depending on the constituent material in which a mode is predominantly concentrated, spin waves exhibit distinct frequency shifts, resulting in a reshuffling of their order in the spectrum. Consequently, in squeezed MCs, different pairs of modes couple compared with the base structure. This opens the possibility—within certain parameter ranges—of designing which pairs will couple within a given field interval and at what field values hybridisation will occur.

In the present paper, we investigate mode hybridisation in two-dimensional Py/Co MCs composed of permalloy dots embedded in a cobalt matrix. We restrict our analysis to structures feasible with current fabrication technologies and employ the plane-wave method (PWM) to compute spin-wave spectra. Our focus is on the effects accompanying hybridisation and the conditions under which it emerges. A key element of the analysis is the exchange of the concentration factor between hybridising modes. This parameter describes the fraction of spin-wave energy residing in a particular constituent material. Its exchange between modes reflects an energy transfer between the components of the composite and provides a means of identifying hybridisation even when other signatures—such as anti-crossing or profile interchange—are barely detectable. We also show cases in which no clear swapping of spatial mode profiles occurs, while a distinct exchange of concentration factors still takes place.

## The model and the method

**Fig. 1:**
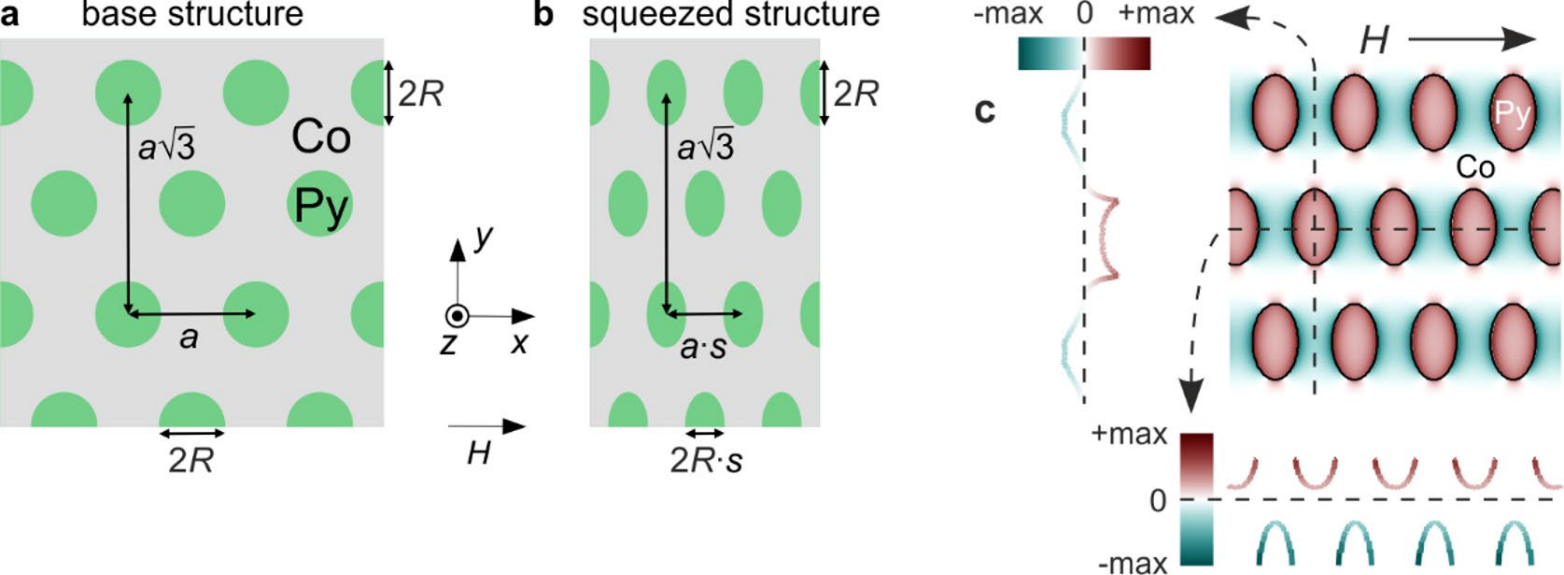
Sketch of a 2D MC based on a hexagonal lattice: a thin-film cobalt matrix with embedded permalloy dots. (**a**) Base structure – hexagonal lattice. (**b**) Squeezed structure – the entire *x*-dimension compressed by a factor *s*. (**c**) Demagnetising field in the squeezed Py/Co MC together with its cross-sectional profiles parallel (bottom) and perpendicular (left) to the external field.

In this study, we analyse the spin-wave spectra of two-dimensional magnonic crystals consisting of a thin cobalt (Co) film forming the matrix, with embedded permalloy (Py) dots. The dots form a hexagonal lattice with a lattice constant of *a* = 600 nm (Fig. 1a). Each dot has a diameter of 340 nm, and the overall film thickness is fixed at 30 nm. The material parameters used in the calculations fall within ranges reported in the literature^63,67–71^: the saturation magnetisation is 1.390e6 A/m for Co and 0.810e6 A/m for Py, while the exchange-stiffness constants are 2.8e-11 J/m for Co and 1.1e-11 J/m for Py. An external magnetic field *H* is applied in-plane along the *x*-axis. The field range considered (250–75 mT) ensures magnetic saturation. This is confirmed by our results: all frequencies in the spectrum remain well above zero, indicating the absence of Goldstone modes associated with remagnetisation processes.

At the Co/Py interface, the magnetisation contrast gives rise to a non-uniform demagnetising field (Fig. 1c)^72,73^. As shown in our earlier work, this field strongly affects the spin-wave spectrum. One way to enhance its influence is by compressing the magnonic crystal along the direction of the external magnetic field^27^. Accordingly, we also consider a squeezed structure (Fig. 1b), in which the *x*-dimension is reduced by a factor *s*, hereafter referred to as the structure ratio. The base structure corresponds to *s* = 1.0, while *s* < 1 denotes compression.

Spin-wave spectra and mode profiles were computed using the plane-wave method (PWM), based on the Landau–Lifshitz equation without damping, as damping has a negligible effect on mode frequencies. This approach is well established and extensively described in the literature^71,74–77^.

A key parameter in our analysis is the concentration factor, *cf*, introduced in^73^. It quantifies the preferential localisation of spin-wave excitations in one constituent material relative to the other, or equivalently, the fraction of the total spin-wave energy concentrated in a given component of the magnonic crystal. For cobalt, it is defined as:

1 $$\mathrm{c}\mathrm{f}=\frac{{\stackrel{\sim}{\mathrm{m}}}_{\mathrm{C}\mathrm{o}}}{{\stackrel{\sim}{\mathrm{m}}}_{\mathrm{C}\mathrm{o}}+{\stackrel{\sim}{\mathrm{m}}}_{\mathrm{P}\mathrm{y}}}$$

where (${\stackrel{\sim}{\mathrm{m}}}_{\mathrm{C}\mathrm{o}}$) and (${\stackrel{\sim}{\mathrm{m}}}_{\mathrm{P}\mathrm{y}}$) denote the mean-squared amplitudes of the dynamic magnetisation within the Co matrix and Py dots, respectively.

It is important to distinguish concentration from localisation. Localisation refers to the spatial character of the wave function—typically involving exponential decay of the amplitude—and may shift the corresponding energy level into the band gap^78,79^. In our work, we do not analyse localisation. Instead, we focus on determining in which constituent material a given mode is concentrated; that is, how the excitation energy is distributed between the components of the MC. The in-Co concentration coefficient cf., defined in Eq. (1), is well suited for capturing this distribution. A value *cf* > 0.5 indicates that the mode is predominantly excited in Co, while *cf* = 1 corresponds to excitation entirely confined to cobalt. Conversely, values approaching zero signify concentration in permalloy.

## Results and discussion

**Fig. 2:**
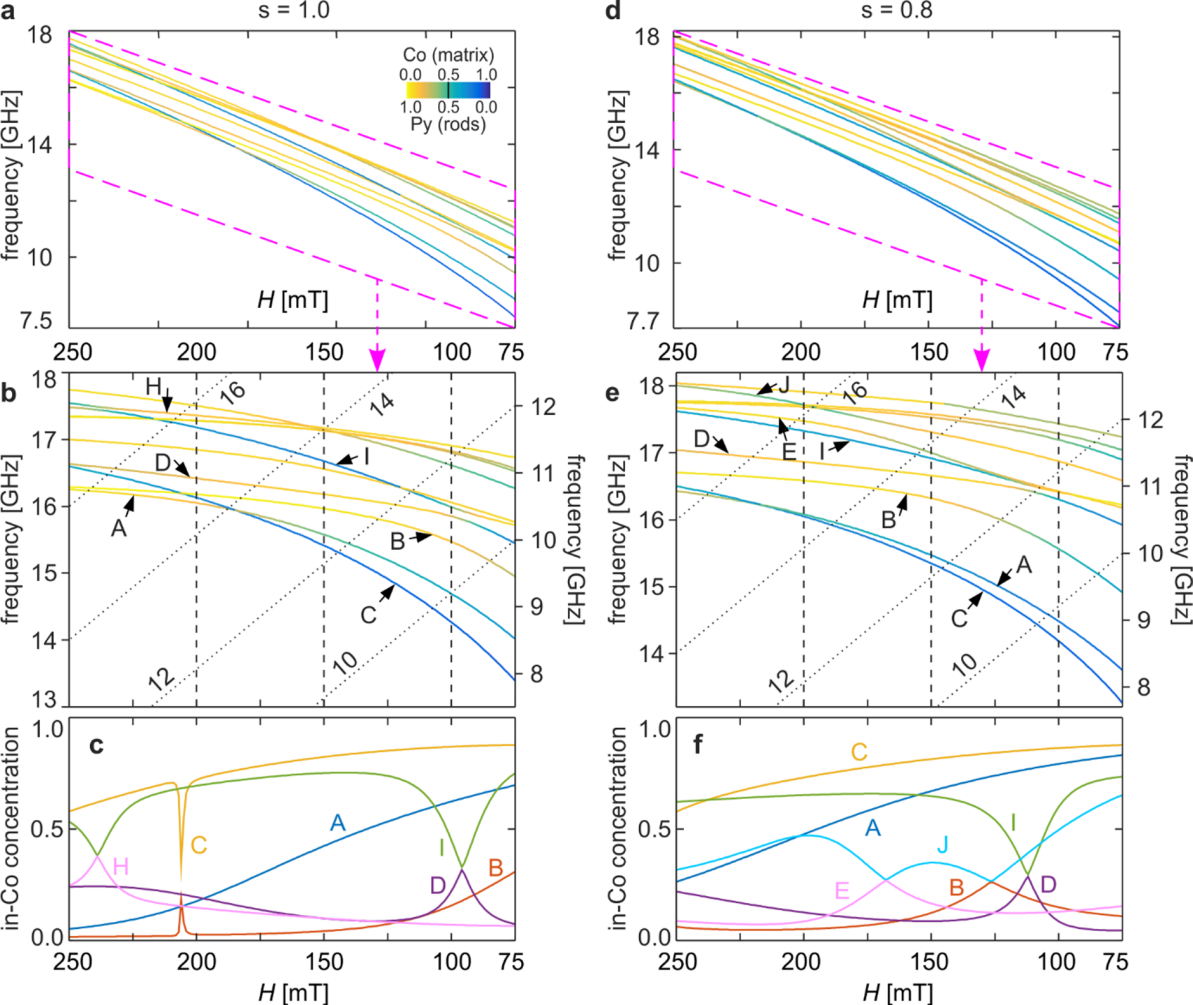
Evolution of the spin-wave spectrum at the centre of the first Brillouin zone (*k* = 0) in a two-component Py/Co magnonic crystal as the external magnetic field *H* varies from 250 to 75 mT. Panels (**a**–**c**) correspond to the base structure (*s* = 1.0), and panels (**d**–**f**) to the compressed structure (*s* = 0.8). (**a**, **d**) Ten lowest spin-wave frequencies plotted as functions of *H*. The line colours indicate the concentration coefficient in Co, calculated using Eq. (1), according to the colour scale shown in the inset. (**b**, **e**) Enlarged view of the regions marked by the purple parallelograms in panels (**a**) and (**d**). The frequency axis is tilted proportionally to the variation of *H*, and lines of constant frequency are shown as diagonal dashed lines. (**c**, **f**) Dependence of the in-Co concentration coefficient on *H* for selected modes labelled A–F, according to their order in the spectrum at *H* = 250 mT for the base structure.

Figure 2 summarizes the evolution of the spin-wave spectrum at the centre of the first Brillouin zone (*k* = 0) as a function of the external magnetic field *H*. Panels 2a–c correspond to the base structure (*s* = 1.0), while panels 2d–f show the compressed structure (*s* = 0.8). Panels 2a and 2d display the ten lowest spin-wave frequencies in the field range 250–75 mT. The colour of each branch represents the concentration factor in cobalt, defined in Eq. (1). To resolve fine spectral features that are not clearly visible on the full scale, panels 2b and 2e show enlarged regions of the spectra with a tilted frequency axis; lines of constant frequency are shown as diagonal dashed lines. The corresponding field dependence of the concentration coefficient for selected modes (labelled A–F) is presented in panels 2c and 2 f. This coefficient reflects the fraction of the excitation energy residing in cobalt. A value around 0.5 indicates an even distribution between Co and Py, values approaching unity denote dominant concentration in Co, and values close to zero correspond to modes concentrated in Py.

For both structures, all spin-wave frequencies decrease as the external field is reduced. At high fields, most modes are predominantly concentrated in permalloy (yellow), whereas a smaller number of modes exhibit strong concentration in cobalt (blue). These Co-dominated modes soften significantly faster than Py-dominated ones, leading to a reordering of the spectrum as the field decreases. In addition, several modes gradually change their concentration character from Py-dominated to Co-dominated, as reflected by a continuous increase of the in-Co concentration factor.

This behaviour originates from the demagnetising field at the interfaces between the Py dots and the Co matrix. The effective magnetic field governing spin-wave precession consists of the external field, the material-dependent contribution related to the saturation magnetisation, and the demagnetising field arising from magnetisation contrast at the interfaces^72,73^. In the present geometry (Py dots embedded in a Co matrix), the demagnetising field reduces the effective field in the Co regions between the dots while enhancing it inside the Py dots. As a result, the Co matrix becomes energetically favourable for low-frequency spin-wave excitation, particularly at low external fields. This leads to a progressive transfer of excitation energy from Py into Co, manifested as an increase in the in-Co concentration factor and an accelerated softening of Co-dominated modes, as external field decreases.

Compression of the magnonic crystal along the field direction modifies this behaviour in two competing ways. On the one hand, the reduced available space for spin-wave formation leads to an overall increase in excitation frequencies, which is most pronounced for Py-dominated modes. On the other hand, compression enhances the demagnetising field^80^, further favouring spin-wave concentration in the Co matrix. Consequently, Co-dominated modes exhibit little frequency increase under compression and may even soften more strongly at low fields, as reflected by their larger concentration factors in Fig. 2f.

We now briefly analyse the characteristic evolution of selected individual modes. For the base structure (*s* = 1.0) at *H* = 250 mT, the two lowest-frequency modes, A and B, are predominantly concentrated in permalloy, with *cf(A)* = 0.05 and *cf(B)* = 0.02. As the external field is reduced, mode A exhibits a monotonic increase of the in-Co concentration factor, leading to a progressively stronger softening and a nonlinear dependence of its frequency on *H*. As a result, mode A gradually separates from the rest of the spectrum.

In contrast, the concentration factor of mode B remains nearly constant down to approximately 150 mT, apart from a narrow peak around 206 mT. Correspondingly, its frequency decreases almost linearly with the field at higher *H*, while a more pronounced softening sets in at lower fields, similar to the behaviour observed for mode A.

Mode C, which is the third mode in the spectrum at high field, is already significantly concentrated in cobalt at *H* = 250 mT (*cf* = 0.58). With decreasing field, its in-Co concentration increases further, resulting in a much faster reduction of its frequency compared to modes A and B. Consequently, mode C moves downward in the spectrum, becoming the second-lowest mode below approximately 206 mT and the lowest-frequency mode below about 185 mT. At weaker fields, the concentration factor increases only slightly, while the frequency continues to decrease rapidly, indicating that once a sufficiently high in-Co concentration is reached, further increases of cf. are not required to sustain strong softening.

Around *H* ≈ 206 mT, a sharp increase of the concentration factor for mode B coincides with a pronounced decrease for mode C, occurring at the field where the two modes exchange their order in the spectrum. This behaviour indicates profile mixing, i.e. mode hybridisation, although the associated anti-crossing remains weak and is barely discernible even in the enlarged view shown in Fig. 2b. In contrast, the exchange of order between modes C and A at approximately 185 mT is not accompanied by such effects: the corresponding frequency branches intersect rather than repel each other, indicating the absence of hybridisation between these modes.

**Fig. 3:**
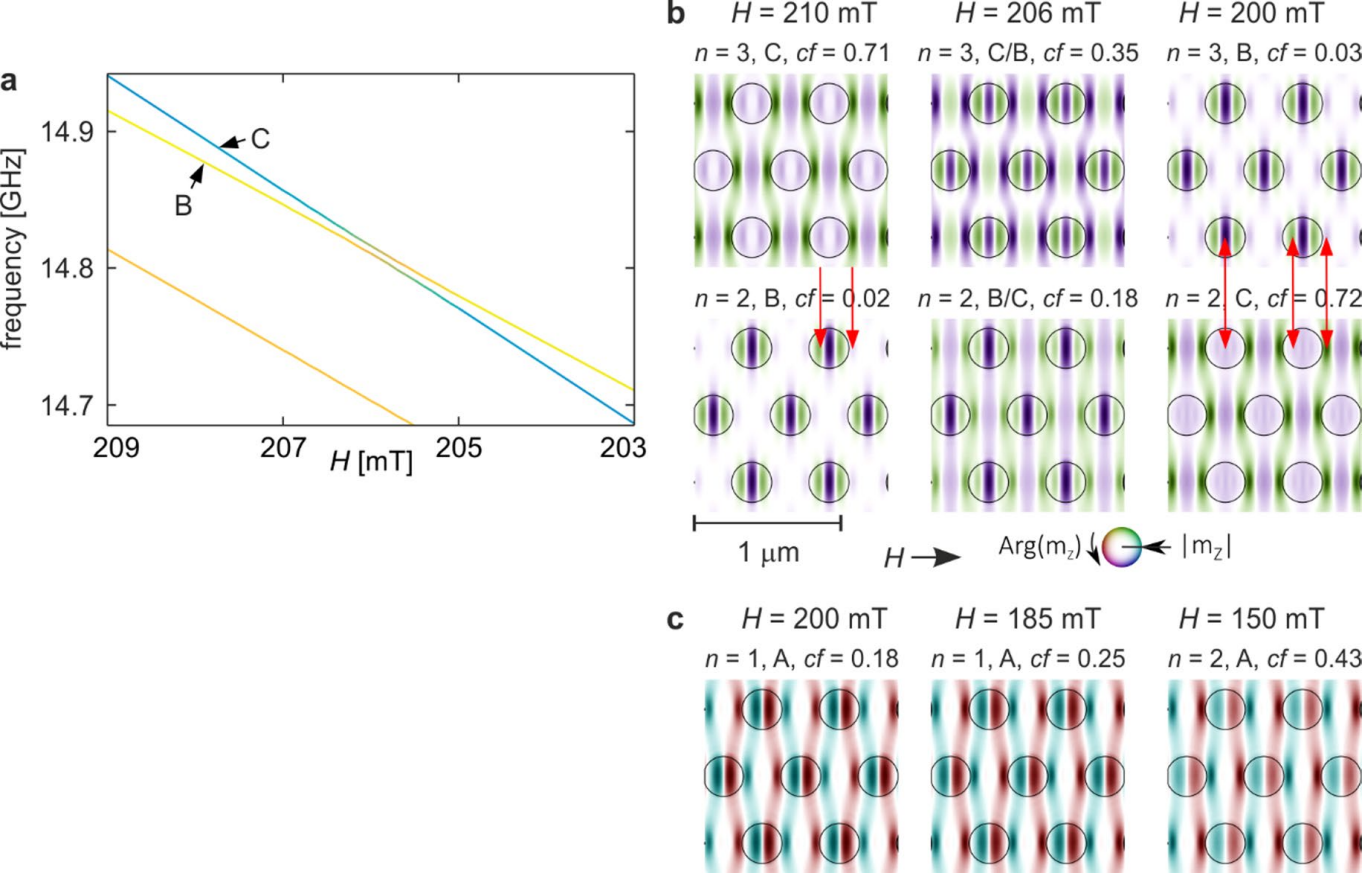
Hybridisation of modes B and C from Fig. 2a–c for the base structure (*s* = 1.0). (**a**) Enlarged fragment of Fig. 2a showing the evolution of the two modes in the field range 209–203 mT. The frequencies exhibit a clear anti-crossing, while the line colours indicate the in-Co concentration coefficient as in Fig. 2a. (**b**) Evolution of the corresponding mode profiles across the hybridisation region. The three columns represent, respectively: a field above the hybridisation point (left), the field at which hybridisation is strongest (centre), and a field below the coupling region (right). Colours denote the phase, and intensity denotes the amplitude of the dynamic magnetisation. Circles mark the boundaries of the Py dots. Red arrows indicate pairs of corresponding amplitude maxima that enable profile matching and hence mode coupling. (**c**) Mode A profile evolution across the region of the frequency crossing with mode C.

Figure 3 illustrates the hybridisation between modes B and C identified in Fig. 2a–c for the base structure (*s* = 1.0). Panel 3a shows an enlarged fragment of the spectrum in the field range 209–203 mT, where the two modes approach each other to within approximately 5 MHz but do not cross, forming a clear avoided crossing. This spectral repulsion is accompanied by an exchange of the in-Co concentration between the modes, visible in Fig. 2c as sharp, oppositely signed features in the *cf(H)* dependencies of modes B and C. The hybridisation occurs within a very narrow field interval of about 1 mT, which explains why it is barely discernible in the full spectrum.

The evolution of the mode profiles across the coupling region is shown in Fig. 3b. The three columns correspond to magnetic fields before, at, and after the hybridisation point. In each column, the profiles are ordered according to their position in the frequency spectrum, with the lower-frequency mode shown below the higher-frequency one. Numerical mode indices (*n*) indicate the ordering in the spectrum, while alphabetical labels (B, C) denote the characteristic profile type. Consequently, when hybridisation leads to an exchange of spectral order, the profile labels swap between the two branches, directly reflecting the physical reordering of the modes.

Before hybridisation, mode C corresponds to the higher-frequency branch (*n* = 3) and mode B to the lower one (*n* = 2). At the coupling point, the profiles exhibit pronounced mixing, with features of both modes appearing in each branch. After hybridisation, the modes recover their characteristic profiles, but their spectral order is reversed: the profile originally associated with mode C becomes the *n* = 2 mode, while profile B shifts to *n* = 3. This exchange is quantitatively confirmed by the in-Co concentration factors shown next to each profile, which swap between the two modes across the hybridisation region and deviate strongly at the coupling point. These deviations are the direct origin of the sharp cf. peaks observed in Fig. 2c.

This case displays all characteristic signatures of mode hybridisation: (i) an avoided crossing of the spectral branches, (ii) an exchange of the concentration factor between the modes, and (iii) strong mixing and swapping of mode profiles. The analysis of the concentration factor therefore provides a sensitive indicator of hybridisation, particularly in situations where the small frequency separation makes the avoided crossing difficult to resolve directly.

The structure of the mode profiles reveals the physical conditions enabling coupling. Prior to hybridisation, mode C exhibits a multi-nodal phase pattern along the field direction, with phase variation occurring predominantly in the Co matrix and a uniform phase inside the Py dots, corresponding to the fundamental mode^26,81,82^ with a noticeable suppression of precession at the centre. In contrast, mode B is concentrated mainly within the Py dots and displays a nodal structure inside each dot, while only weak amplitudes are present in the matrix. Despite these differences, the spatial arrangement of dominant amplitude maxima for mode C matches the maxima for mode B (red arrows in Fig. 3b).

This spatial compatibility of antinodes constitutes the key condition for coupling. At the hybridisation point, both modes form superpositions of the original profiles, combining dot-centred features characteristic of mode B with matrix-dominated features characteristic of mode C, with a relative phase shift between the two hybridised states. After passing through the coupling region, the modes largely recover their original character, apart from minor residual modifications. The persistent alignment of corresponding amplitude maxima before, during, and after hybridisation confirms that profile compatibility—specifically the matching of antinodal regions—is the physical basis for mode coupling in this system^27^.

Figure 3c illustrates the evolution of mode A’s profile. The columns correspond to fields before, at, and after the crossing of its frequency branch with mode C. At the field where the frequencies intersect, the profile of mode A remains essentially unchanged, indicating the absence of hybridisation. This behaviour arises from the spatial symmetry mismatch: mode A is antisymmetric within both the matrix and the dots, whereas mode C is symmetric. The incompatibility of their profiles prevents coupling between these modes.

Returning to Fig. 2, the evolution of mode D exhibits behaviour distinct from that seen for modes A–C. Below approximately *H* ≈ 240 mT, its in-Co concentration decreases rather than increases. As a result, its frequency dependence *f(H)* remains nearly linear down to the hybridisation region with mode I near *H* ≈ 96 mT. This observation reinforces the conclusion drawn earlier: changes in the concentration factor play a decisive role in determining the rate at which the spin-wave frequency decreases with the external magnetic field.

**Fig. 4:**
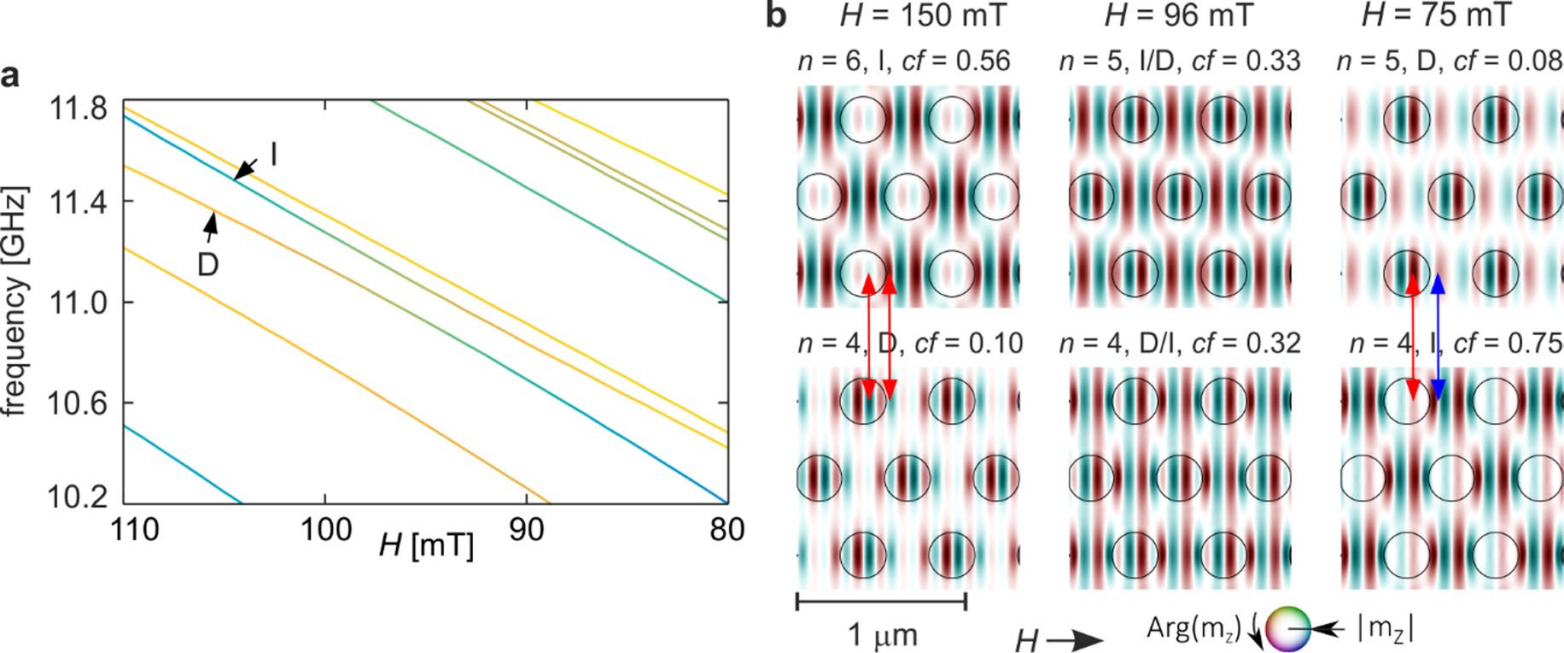
Hybridisation of modes D and I from Fig. 2a–c for the base structure (*s* = 1.0). (**a**) Enlarged fragment of Fig. 2a showing the evolution of the two modes in the field range 110–80 mT. The line colours indicate the in-Co concentration coefficient, as in Fig. 2a. (**b**) Evolution of the spatial profiles of the two hybridising modes. The three columns correspond to fields above the hybridisation (left), at the point of strongest hybridisation (centre), and below it (right). Colours represent the phase and intensity the amplitude of the dynamic magnetisation. Circles mark the boundaries of the Py dots. Red arrows highlight corresponding amplitude maxima that indicate profile matching between the two modes.

Figure 4 illustrates the hybridisation between modes D and I identified in Fig. 2. Panel 4a shows an enlarged fragment of the spectrum in the field range 125–75 mT, where the two branches approach one another but with a weaker avoided crossing than observed for modes B and C. Despite this, the exchange of in-Co concentration between the modes is pronounced, visible as positive peaks for mode D and negative peaks for mode I in Fig. 2c. The coupling extends over a broad field interval of approximately 50 mT, with the minimum frequency separation around 130 MHz.

The evolution of the mode profiles across the hybridisation region is shown in Fig. 4b. The three columns correspond to magnetic fields before (*H* = 150 mT), at (*H* ≈ 96 mT), and after (*H* = 75 mT) the coupling point. In each column, profiles are ordered by frequency, with the lower-frequency mode displayed below the higher-frequency one. Hybridisation leads to a swap in spectral order: before coupling, mode D corresponds to *n* = 4 and mode I to *n* = 6, whereas after hybridisation D shifts to *n* = 5 and I to *n* = 4.

Before hybridisation, mode I exhibits a multi-nodal phase pattern along the *x*-direction in the matrix, with three nodal lines inside the dots and four in the inter-dot regions, forming six alternating-phase regions. Along the *y*-direction, phase reversals occur when moving from matrix to dot, whereas the matrix itself shows uniform phase in non-zero amplitude paths. Mode D displays nearly uniform phase along *y*, with amplitude minima between rows, and eight alternating-phase regions along *x* separated by nine nodal lines, including two inside the dots.

At the hybridisation field, the upper (I/D) profile combines the matrix excitation characteristic of I with enhanced amplitude inside the dots, resembling D, with an overall phase inversion. The lower (D/I) profile similarly exhibits a superposition of dot-centered excitation from D and matrix-dominated features from I, with reversed phase in the matrix. This superposition generates additional alternating-phase regions and nodal lines along *x*, with six and eight alternating regions for I/D and D/I, respectively. Red arrows in Fig. 4b indicate pairs of corresponding amplitude maxima, highlighting spatial alignment before, during, and after hybridisation.

After hybridisation, both modes largely recover their characteristic profiles, but not entirely. Mode D restores its pre-hybridisation form inside the dots but shows a reduced number of alternating-phase regions in the matrix, similar to the original I profile. Conversely, mode I recovers its matrix structure but exhibits inverted phase inside the dots, leading to adjacent dot–matrix regions sharing the same phase sign.

These observations demonstrate that hybridisation between D and I produces branch repulsion, concentration exchange, profile mixing, and qualitative transformations of mode profiles. The increase in nodal-line count along *x* above the hybridisation point is consistent with previous reports for two-dimensional nanodot arrays in the vortex state^26^. The persistent alignment of amplitude maxima confirms that spatial compatibility of antinodes underlies the coupling mechanism in this system.

**Fig. 5:**
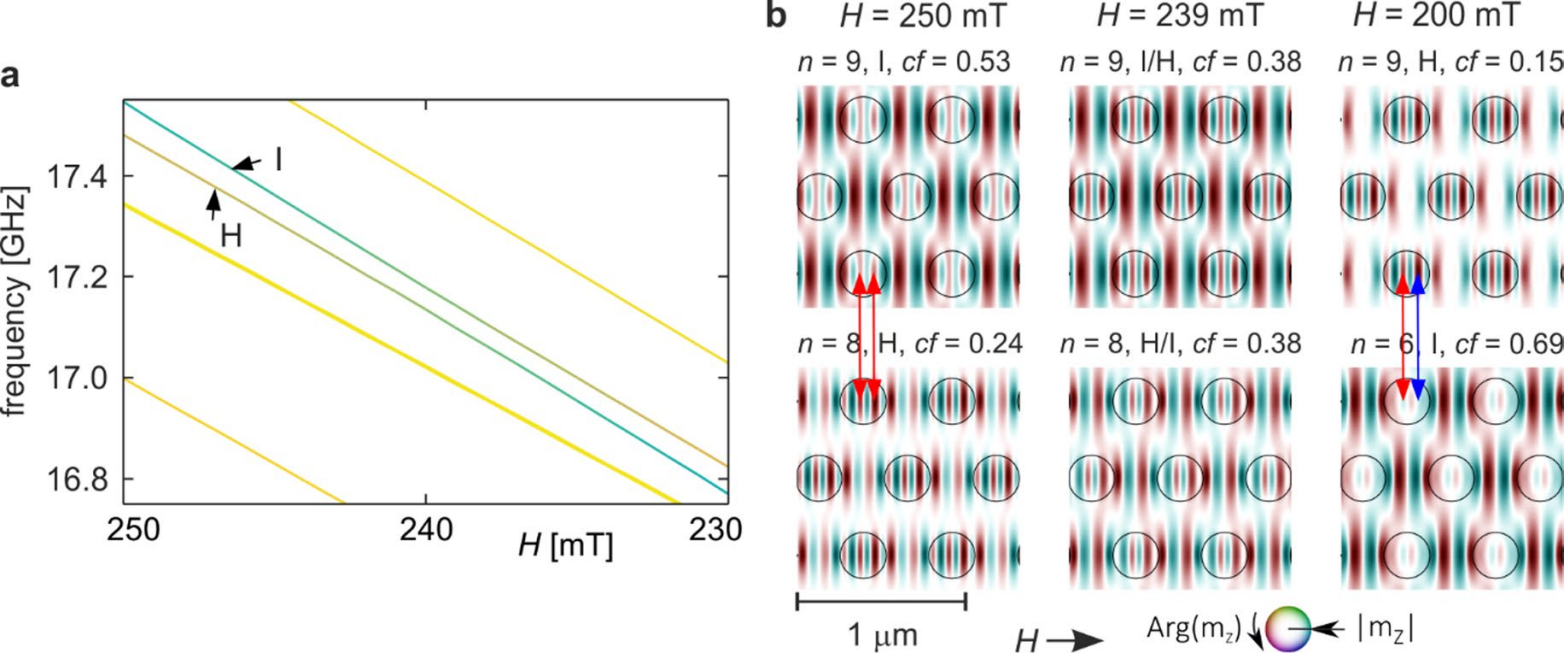
Hybridisation of modes H and I from Fig. 2a–c for the base structure (*s* = 1.0). (**a**) Enlarged fragment of Fig. 2a showing the evolution of the two modes in the field range 250–230 mT. Line colours indicate the in-Co concentration coefficient, as in Fig. 2a. (**b**) Evolution of the spatial profiles of the hybridising modes. Columns correspond to fields above the hybridisation (left), at maximum coupling (centre), and below it (right). Colours denote the phase, and intensity the amplitude of the dynamic magnetisation. Circles indicate the boundaries of the Py dots. Red arrows mark pairs of corresponding amplitude maxima that facilitate the coupling between the two modes.

Mode I also hybridises with another mode, denoted H in Fig. 2a–c, within a narrow field interval around *H* ≈ 239 mT. Although this hybridisation is only weakly visible in the frequency spectrum (Fig. 2b), it is clearly manifested in the in-Co concentration coefficient (Fig. 2c), appearing as a prominent positive peak for mode H and a corresponding negative peak for mode I. An enlarged view of this spectral region is shown in Fig. 5a. As in previous cases, the two branches repel each other, albeit with a weaker effect. Nevertheless, a distinct exchange of the in-Co concentration coefficient occurs, with a smaller *cf* contrast than observed for the D–I or B–C hybridisations. The coupling spans approximately 25 mT, with a minimum frequency separation of about 43 MHz.

The spatial profiles of modes H and I across the hybridisation region are shown in Fig. 5b. Mode H resembles mode D (Fig. 4b). Before hybridisation (left column, lower profile), its structure in the cobalt matrix is similar to D, though with a stronger excitation amplitude (*cf* = 0.24). Inside the permalloy dots, an additional pair of local maxima is present. Below the hybridisation point (right column, upper profile), the profile maintains this pattern, reflecting field-dependent modifications.

Mode I is the same mode that previously hybridised with mode D (Fig. 4), now observed at a higher field range. Above its coupling with mode H (left column, upper profile), it exhibits an additional pair of local maxima inside the Py dots. At the hybridisation field (*H* = 239 mT, middle column), both profiles display the characteristic features of mode coupling: (i) mixing and interchange of profile characteristics, (ii) repulsion of spectral branches, and (iii) exchange of concentration factors. After passing through the hybridisation region, the modes partially recover their pre-hybridisation features, with modifications in the number and placement of amplitude maxima.

For mode I, the dot profiles are analogous to those observed during its hybridisation with mode D. As the field decreases below the hybridisation point, the matrix pattern is largely preserved, while the number of maxima inside the dots reduces from three to two. Conversely, mode H exhibits minimal changes inside the dots, but an amplitude maximum in the matrix disappears after hybridisation. These modifications are highlighted in Fig. 5b by red arrows. Above the hybridisation field, two corresponding maxima are visible for both modes, aligned in position but opposite in phase. Below the hybridisation, only one such matching maximum remains; at *H* = 200 mT, a blue arrow marks a maximum of mode H corresponding to a nodal line of mode I, whereas at *H* = 250 mT, the same location corresponded to a local maximum of mode I.

Thus, as in earlier examples, hybridisation between modes H and I produces a qualitative transformation of the profiles, reorganising local maxima and nodal lines, modifying phases, and altering the distribution of excitation amplitudes inside dots and matrix regions. Despite the weaker anti-crossing, the exchange of the concentration factor and the field-dependent inversion of amplitude structure leave no doubt that substantial hybridisation occurs.

**Fig. 6:**
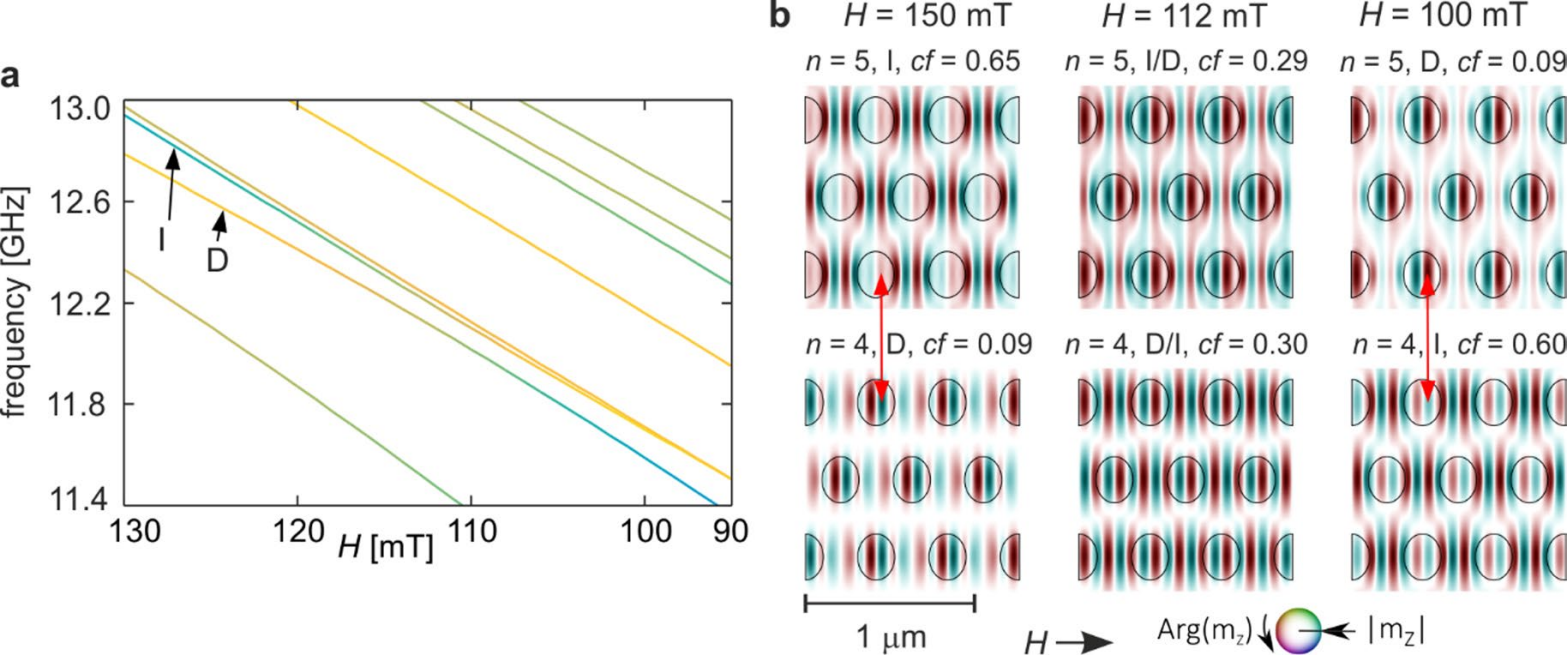
Hybridisation of modes D and I from Fig. 2d–f for the compressed structure (*s* = 0.8). (**a**) Enlarged fragment of Fig. 2d showing the evolution of the two modes in the external-field range 130–90 mT. The line colours represent the in-Co concentration coefficient, as in Fig. 2d. (**b**) Evolution of the spatial profiles of the hybridising modes. Columns correspond to fields above the hybridisation (left), at maximum coupling (centre), and below it (right). Colours denote the phase and intensity the amplitude of the dynamic magnetisation. Circles outline the boundaries of the Py dots. Red arrows indicate corresponding amplitude maxima that facilitate coupling between the modes.

The hybridisation of modes D and I in the compressed structure (*s* = 0.8) is shown in Fig. 6. Panel 6a presents an enlarged portion of the spectrum from Fig. 2d, while panel 6b displays the evolution of the profiles of the coupled modes. The two modes interact within the external-field range of approximately 120–100 mT, and the minimum frequency separation between them is about 83 MHz. As in the case of the base structure (*s* = 1.0), the hybridisation is characterised by three hallmark effects: (i) repulsion of the frequency branches, (ii) an exchange of the concentration factor in cobalt, and (iii) the swapping of the order of the mode profiles. Corresponding amplitude maxima, marked by red arrows in Fig. 6b, confirm the spatial matching required for hybridisation.

Mode D undergoes changes in the number of local amplitude maxima analogous to those observed in the base structure. Along the horizontal segment connecting the centre of a Py dot to the midpoint between neighbouring dots (the *x*-direction), at higher fields (left column of Fig. 6b), three amplitude maxima appear with alternating phases. At lower fields (right column), the maxima inside the dot and at the dot boundary become phase-aligned and merge into a single, stronger maximum above the dot. Mode I displays complementary behaviour: at lower fields an additional amplitude maximum appears inside the dot, with opposite phase to the maximum at the boundary.

For comparison, the profiles of these two modes in the base structure (*s* = 1.0) at *H* = 150 mT are shown in Fig. 4b (left column). This corresponds to the same external field used for the compressed structure in Fig. 6b. The profile of mode I (upper-left panels in both figures) reveals that compression does not significantly alter the structure of the excitation in the matrix. However, inside the dots, a phase inversion occurs: each half of the dot becomes approximately uniformly phased, and the number of nodal lines decreases from three to one. Remarkably, this is the same effect previously observed for mode I (*s* = 1.0) after passing through hybridisation.

Mode D in the compressed structure above the hybridisation region (lower-left panel of Fig. 6b) exhibits the same qualitative features as mode D in the base structure below the hybridisation region (upper-right panel in Fig. 4b). After hybridisation at *s* = 0.8, however, mode D acquires additional features distinct from those in the uncompressed structure, demonstrating that squeezing amplifies or modifies the effects associated with hybridisation.

**Fig. 7:**
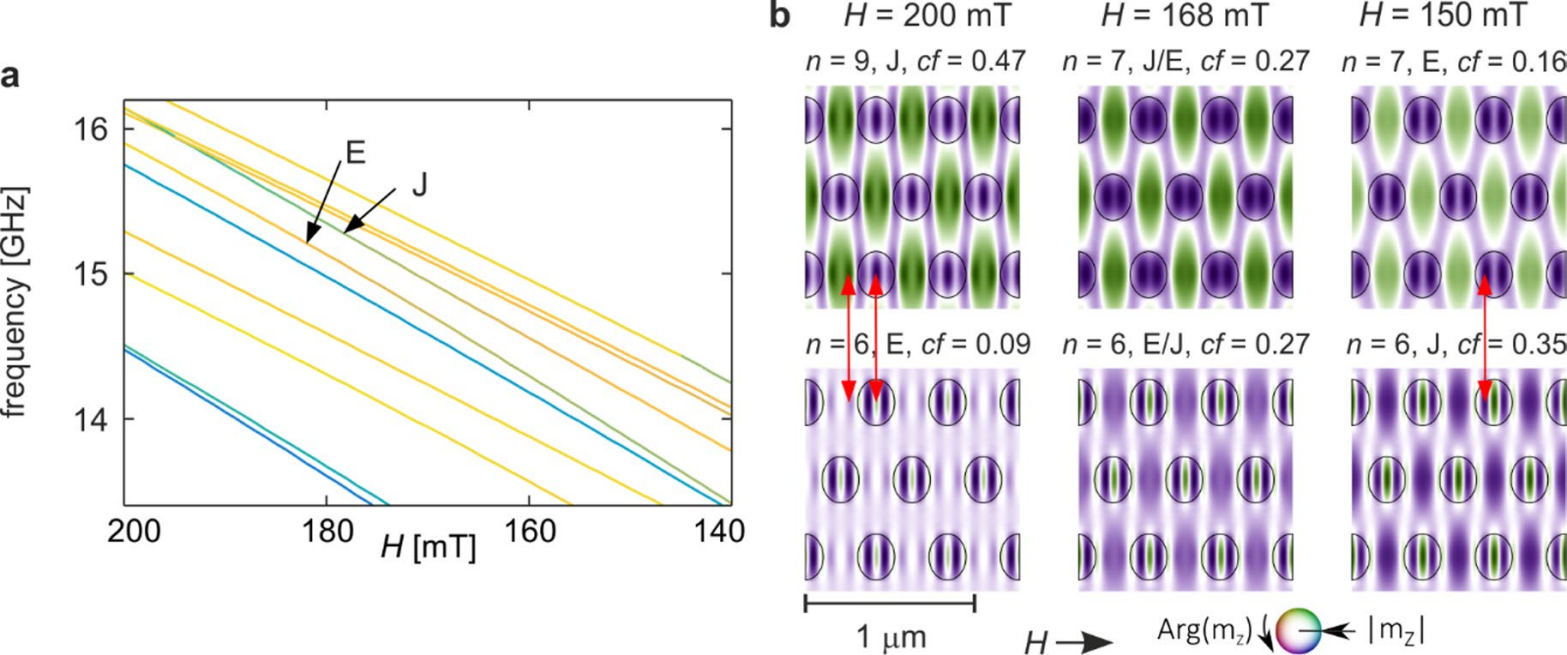
Hybridisation of modes E and J from Fig. 2d–f for the compressed structure (*s* = 0.8). (**a**) Enlarged fragment of Fig. 2d showing the evolution of the two modes in the external-field range 200–140 mT. Line colours indicate the in-Co concentration coefficient, as in Fig. 2d. (**b**) Evolution of the spatial profiles of the hybridising modes. Columns correspond to field values above the hybridisation (left), at maximum coupling (centre), and below it (right). Colours denote the phase, and intensity the amplitude of the dynamic magnetisation. Circles indicate the boundaries of the Py dots. Red arrows highlight corresponding amplitude maxima that underpin the coupling between modes E and J.

**Fig. 8:**
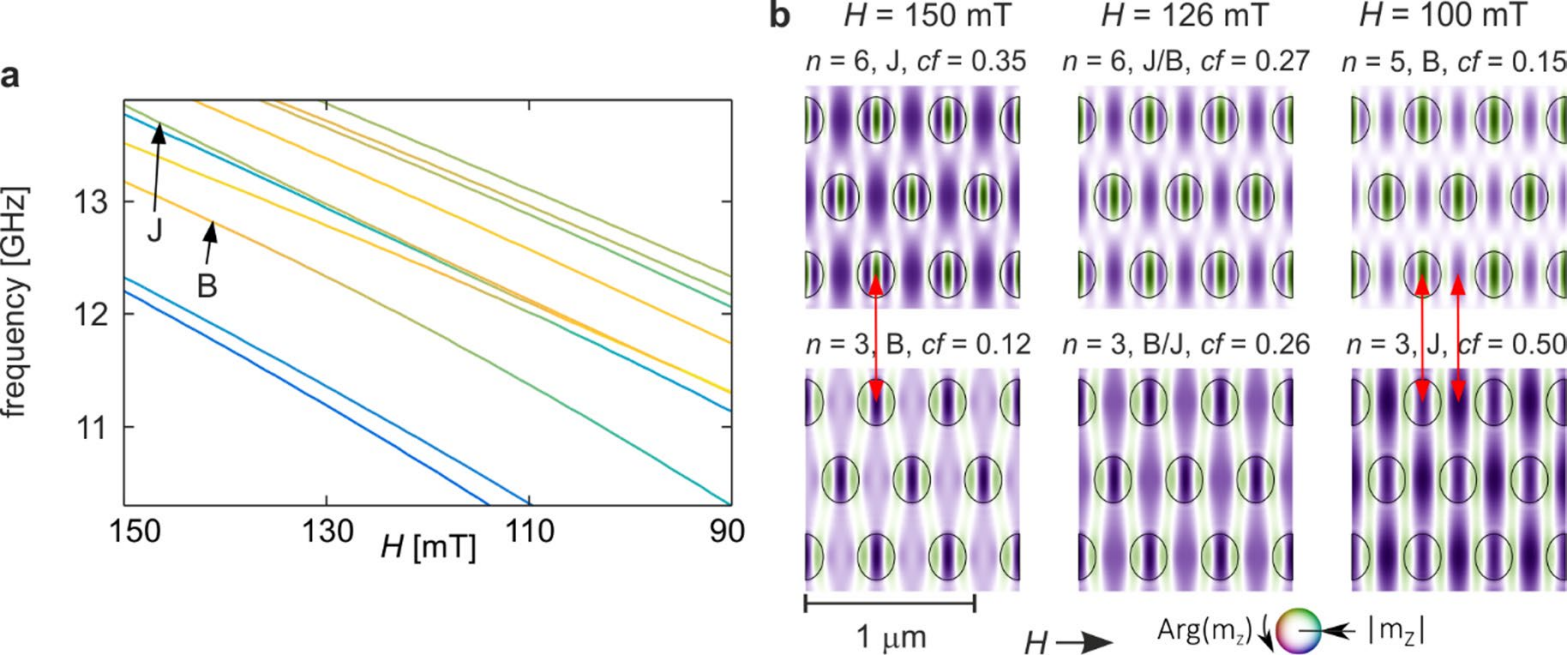
Second hybridisation of mode J, this time with mode B, from Fig. 2d–f for the compressed structure (*s* = 0.8). (**a**) Enlarged fragment of Fig. 2d showing the evolution of the two modes in the external-field range 150–90 mT. Line colours indicate the in-Co concentration coefficient, as in Fig. 2d. (**b**) Evolution of the spatial profiles of the hybridising modes. Columns correspond to fields above the hybridisation (left), at maximum coupling (centre), and below it (right). Colours denote the phase and intensity the amplitude of the dynamic magnetisation. Circles indicate the boundaries of the Py dots. Red arrows highlight corresponding amplitude maxima that allow coupling between modes B and J.

Mode J undergoes two distinct hybridisation events in the compressed structure (*s* = 0.8): first with mode E, and later with mode B (the latter discussed in Fig. 8). The first of these is illustrated in Fig. 7. Panel 7a shows an enlarged fragment of the spectrum from Fig. 2d, while panel 7b presents the evolution of the spatial profiles of the hybridising modes.

In the spectrum, the repulsion between the two branches is clearly visible, accompanied by an exchange of the in-Co concentration factor. Although the contrast in the concentration factor is smaller than in the hybridisations discussed previously, the effect remains pronounced and is confirmed by the characteristic peaks in *cf(H)* shown in Fig. 2f—negative for mode J and positive for mode E. The hybridisation occurs within the field interval of approximately 200–150 mT, with a minimum frequency separation of about 233 MHz.

The spatial profiles of the coupled modes exhibit the expected matching of amplitude maxima. At fields above the hybridisation point (200 mT; left column of Fig. 7b), the profiles of both modes display two local amplitude maxima located in the same regions of the structure (marked by red arrows). At lower fields (150 mT; right column), neither of these maxima appears in the same form. The maximum located in the matrix remains visible only in the upper profile (and even there only weakly), whereas in the lower profile it disappears entirely—the double maximum characteristic of mode *n* = 6 at higher fields reduces to a single maximum between neighbouring dots at lower fields. The second maximum, located at the centre of the dot, also undergoes a reorganisation: it remains in the lower profile but disappears from the upper one.

The upper mode (labelled *n* = 7) evolves such that, at lower fields, it exhibits two internal maxima within the Py dot instead of three as at higher fields, although in both cases all maxima share the same phase. These two maxima shift towards the dot centre, aligning with the positions of two maxima of the lower mode, as indicated by the red arrows in the right column of Fig. 7b. Thus, as in earlier examples, hybridisation alters the number and arrangement of amplitude maxima, although here the corresponding maxima have the same phase rather than opposite phase.

In contrast to the earlier hybridisation examples (Figs. 3, 4, 5 and 6), the evolution of the profiles in Fig. 7 does not involve a clear interchange of the characteristic profile shapes. As one moves from left to right across Fig. 7b, the upper mode retains its overall profile shape but loses one internal maximum inside the dot, whereas the lower mode loses one maximum in the matrix, with no obvious exchange of full profile structures. Despite this, the two modes exchange their concentration factors, demonstrating that hybridisation is indeed taking place.

This provides an important insight: the exchange of the concentration factor between hybridising modes does not necessarily require a full swap of their spatial profiles. The modulation of amplitude structure—rather than wholesale exchange of profiles—may be sufficient to induce measurable energy transfer between modes in a composite magnonic system.

This observation becomes particularly meaningful when placed in the broader context of multi-mode interactions: as will be shown in Fig. 8, mode J simultaneously approaches and partially couples with two different modes (E and B) within overlapping field ranges. Such a situation, involving multiple competing couplings, can suppress or distort the clean profile interchange typically expected in a simple two-mode hybridisation. The case shown in Fig. 7 is precisely such an example, where hybridisation manifests primarily through exchange of concentration factor and reorganisation of local amplitude maxima, without a clear, full-profile swap.

The second hybridisation involving mode J occurs with mode B and is shown in Fig. 8. Panel 8a presents an enlarged fragment of the spectrum from Fig. 2d. The repulsion between the two branches is clearly visible, as is the exchange of the concentration factor in cobalt (*cf*), which also confirms the hybridisation of modes D and I examined earlier. The hybridisation between modes B and J takes place within the external-field range of approximately 150–90 mT, with a minimum frequency separation of about 650 MHz.

In contrast to several earlier cases, the higher-frequency mode retains its spatial profile throughout the hybridisation transition (top row of Fig. 8b). The lower-frequency mode also maintains its profile inside the Py dot, so the amplitude maximum in the dot centre remains aligned with the corresponding maximum of the higher-frequency mode, both above and below the hybridisation region.

More notable changes occur in the matrix region between the dots. For fields above the hybridisation point, the amplitude of the lower-frequency mode shows a pronounced decrease at the centre of the inter-dot region, resulting in two distinct local maxima along this segment. Below the hybridisation, this structure collapses into a single maximum positioned at the centre of the same region. This new maximum aligns closely with a corresponding amplitude maximum in the upper mode. Thus, although the lower mode loses one local maximum, its overall character remains stable, and the hybridisation does not result in a full exchange of mode profiles.

This behaviour parallels that of the E–J hybridisation (Fig. 7), further supporting the conclusion that a complete swap of spatial profiles is not a necessary condition for hybridisation. Instead, the essential markers in these cases are the exchange of the concentration factor, the repulsion of frequency branches, and local reconstruction of amplitude maxima—i.e., the reorganisation of excitation energy in specific regions of the sample.

A comparison of the B-mode profiles between the compressed (*s* = 0.8) and base (*s* = 1.0) structures further highlights the role of the demagnetising field. In the compressed structure (Fig. 8b, left column, bottom panel), the amplitude of the spin wave in the matrix is considerably enhanced relative to the base structure (Fig. 3b, left column, bottom panel). This is consistent with the increased *cf* value for Co: compression raises *cf(B)* from 0.02 to 0.12. The strengthening of the demagnetising field due to geometric squeezing makes the cobalt matrix increasingly favourable for spin-wave excitation, shifting the balance of energy concentration between the two constituent materials.

The behaviour of the B–E–J hybridisation therefore raises an important conceptual question: how essential is profile interchange to the definition of hybridisation? In the two final cases (E–J and B–J), all other characteristic hybridisation features are present—branch repulsion, exchange of concentration factor, and matching or reorganised amplitude maxima—yet the profiles do not fully exchange.

A plausible explanation arises from the fact that mode J simultaneously engages in two hybridisation processes within overlapping field ranges. As shown in Fig. 2f, the *cf* curve for mode J first undergoes a sharp drop during hybridisation with mode E; immediately afterwards, before it can return to its monotonic trend, it decreases again as a result of the coupling with mode B. This suggests that in the field interval ~ 160–130 mT mode J interacts concurrently with both modes E and B, giving rise to a three-mode hybridisation scenario.

A similar phenomenon was reported in two-dimensional magnetic dots in the vortex state^26^, where the intermediate-frequency mode retained its profile while the lowest- and highest-frequency modes exchanged theirs. In the present case, the intermediate mode (the one lying spectrally between B and E at given fields) indeed preserves its spatial structure (bottom row of Fig. 7b and top row of Fig. 8b). Meanwhile, the profiles of the outer modes appear to undergo partial exchange—although the similarity of their structures makes this difficult to determine uniquely.

Thus, the hybridisation between B and J represents a complex, multi-mode interaction in which the concentration factor remains a reliable indicator of energy transfer, even when the spatial-profile signature is muted or ambiguous.

## Conclusion

This work has presented a detailed analysis of spin-wave mode hybridisation in two-dimensional Py/Co magnonic crystals composed of permalloy dots embedded in a cobalt matrix. The introduction of an energy-concentration coefficient allowed hybridisation to be identified even in cases where its typical signatures—such as clear branch repulsion or a full exchange of spatial mode profiles—were weakly visible or nearly absent. Our results demonstrate that this parameter serves as a sensitive indicator of mode coupling, providing insight into subtle energy-redistribution processes between the constituent materials of the composite.

We have shown that hybridisation in bi-component magnonic crystals can manifest itself in several forms. These range from the classical scenario involving simultaneous branch repulsion, profile swapping, and concentration-factor exchange, to atypical cases in which no clear profile exchange occurs but a distinct transfer of energy between the materials nevertheless takes place. Of particular interest is the multi-mode hybridisation observed in the compressed structure, in which one mode retains its spatial profile while the other participating modes exchange selected characteristics. Such behaviour highlights the complexity of hybridisation processes in magnonic crystals and reveals mechanisms that go beyond the standard two-mode picture commonly discussed in the literature.

An important aspect of our study has been the comparison between the base and compressed structures. By exploiting the non-uniform dependence of spin-wave frequencies on the external magnetic field, we have shown that compression introduces an additional frequency shift whose magnitude depends on the degree of concentration of a given mode in permalloy or cobalt. Modes predominantly concentrated in Py shift upwards in the spectrum, while those concentrated in Co experience little or even negative shifts at weak fields. Consequently, hybridisation between a Py-concentrated and a Co-concentrated mode occurs at higher external fields in the compressed structure. Compression also causes different modes to come into frequency proximity within a given field interval, thereby giving rise to new hybridising pairs not present in the base structure.

These findings imply that geometric deformation along the external-field direction provides a means of shifting hybridisation to higher field values and of inducing new coupling scenarios. The degree of compression determines not only how far a particular hybridisation is shifted but also which additional pairs of modes exhibit hybridisation within a specific field interval. This opens a route towards the controlled design of magnonic systems in which coupling conditions between selected modes may be tailored through suitable geometric modification. These mechanisms may be used to engineer targeted frequency crossings and tailored coupling regimes, offering new opportunities for magnonic circuitry, filters, and coherent wave-based signal processing.

In summary, our analyses confirm that mode hybridisation in Py/Co magnonic crystals is a multifaceted phenomenon with subtle characteristics that require refined detection tools. The energy-concentration coefficient proves to be particularly effective in revealing hybridisation, even in unconventional cases. The results reported here may be useful for the design of magnonic devices in which controlled coupling between spin-wave modes is essential, such as resonators, switches, directional couplers, and wave-based logic elements.

## Data Availability

Data sets generated during the current study are available from the corresponding author on reasonable request.
